# Highlights and future directions from the European Gynecological Oncology Congress 2017

**DOI:** 10.4274/jtgga.2018.0007

**Published:** 2018-06-04

**Authors:** İlker Selçuk

**Affiliations:** 1 Department of Gynecologic Oncology, Health Sciences University, Zekai Tahir Burak Woman’s Health, Health Application and Research Center, Ankara, Turkey

To the Editor;

The European Gynecological Oncology Congress, 2017, was held in Vienna between November 4^th^-7^th^ with more than 3200 participants and over 1300 abstracts. Some studies will draw perspectives for the future and innovate many researchers in new eras.

Nowadays, less radical cancer surgery is a topic of discussion to prevent morbidities. On the other hand, molecular and genomic research is increasing tremendously to solve the problem completely.

The Lymphadenectomy in Ovarian Neoplasms study by Lorusso et al. (1) evaluated the role of systematic pelvic and paraaortic lymphadenectomy in patients with advanced ovarian cancer. Six hundred forty-seven FIGO IIB-IV patients with epithelial ovarian, fallopian tube or primary peritoneal cancer were randomized in a ratio of 1:1 as those with and without excision of pelvic and paraaortic lymph nodes after macroscopic complete resection of intraperitoneal tumor sites in the absence of bulky lymph nodes pre and intraoperatively. Total operation time, blood loss, number of blood transfusions, re-laparotomy and re-admission rates, and intensive care unit administration were decreased significantly with no lymph node excision (LNE). The median progression-free survival was 26 months after platinum-based chemotherapy in both arms, and the overall survival was 66 and 69 months in the LNE and no-LNE arms, respectively. It is worth reminding that omitting lymphadenectomy in clinically negative lymph nodes improves morbidity and mortality rates in terms of advanced ovarian cancer surgery, without any harm to survival periods.

Cervical cancer is generated by human papilloma virus (HPV) infection and the efficacy of HPV vaccines for the prevention of cervical cancer has previously been documented with bivalent and quadrivalent HPV vaccines. The 9-valent HPV vaccine targets HPV type 6, 11, 16, 18 and 31, 33, 45, 52, 58. Joura (2) randomized 14215 participants to 3 doses of 9-valent HPV vaccine and 3 doses of quadrivalent HPV vaccine. After 6 years of follow-up, incidence of HPV types 6, 11, 16, and 18-related infection, cytologic abnormalities, and treatment rates were similar in both groups; however, HPV types 31, 33, 45, 52, and 58-related infections and cytologic abnormalities were less common in the 9-valent group with an antibody efficacy of 5 years.

Despite efforts to prevent HPV infection before serious high-grade cervical preinvasive lesions, whether high-grade lesions or cervical carcinoma can be treated with vaccines is a matter of research. Park et al. (3) reported the latest study on GX-188E, a DNA therapeutic vaccine encoding for HPV types 16/18 - E6/E7. GX-188E had a successful phase I trial on nine patients, seven of whom had complete regression of cervical intraepithelial neoplasia 3 (CIN3). Sixty-eight patients with confirmed CIN3 and HPV 16 or 18 were randomized to 1 mg or 4 mg GX-188E at weeks 0, 4, and 12 (1:1). At week 20 and 36, 51.5% and 59.4% of patients regressed to CIN1, respectively. Small lesions (<50% of cervix by gross colposcopic evaluation) had better regression rates (78.8% vs 38.7%) and 1 mg of GX-188E had higher rates, albeit not significant, of regression at week 36 when compared with 4 mg of GX-188E (66.7% vs 52.9%).

New HPV vaccines, with regard to prevention and treatment, will have a role in the limitation and possibly the eradication of cervical cancer.

## References

[1] Lorusso D, Sehouli J, Reuss A, Vergote I, Marth C, Kim JW, et al (2017.). LION – lymphadenectomy in ovarian neoplasms. a prospective randomized AGO study group led gynecologic cancer intergroup trial. European Gynaecological Oncology Congress.

[2] Joura E (2017.). Long-term efficacy and immunogenicity of the 9-valent human papillomavirus vaccine: final analyses of a double-blind, randomized clinical study. European Gynaecological Oncology Congress.

[3] Park SH, Lee K, Kim J, Choi Y, Lee S (2017). Therapeutic HPV vaccine-is it clinically useful?. European Gynaecological Oncology Congress 2017.

